# Cyclin B1 is essential for mitosis in mouse embryos, and its nuclear export sets the time for mitosis

**DOI:** 10.1083/jcb.201612147

**Published:** 2018-01-02

**Authors:** Bernhard Strauss, Andrew Harrison, Paula Almeida Coelho, Keiko Yata, Magdalena Zernicka-Goetz, Jonathon Pines

**Affiliations:** 1 The Gurdon Institute, Cambridge, England, UK; 2 Department of Zoology, University of Cambridge, Cambridge, England, UK; 3 Department of Genetics, University of Cambridge, Cambridge, England, UK; 4 Department of Physiology, Development and Neuroscience, University of Cambridge, Cambridge, England, UK; 5 The Institute of Cancer Research, London, England, UK

## Abstract

There is remarkable redundancy between the Cyclin–Cdk complexes that comprise the cell cycle machinery. None of the mammalian A-, D-, or E-type cyclins are required in development until implantation, and only Cdk1 is essential for early cell divisions. Cyclin B1 is essential for development, but whether it is required for cell division is contentious. Here, we used a novel imaging approach to analyze Cyclin B1–null embryos from fertilization onward. We show that Cyclin B1^−/−^ embryos arrest in G2 phase after just two divisions. This is the earliest arrest of any Cyclin known and places Cyclin B1 with cdk1 as the essential regulators of the cell cycle. We reintroduced mutant proteins into this genetically null background to determine why Cyclin B1 is constantly exported from the nucleus. We found that Cyclin B1 must be exported from the nucleus for the cell to prevent premature entry to mitosis, and retaining Cyclin B1–Cdk1 at the plasma membrane precludes entry to mitosis.

## Abstract (precis)

Strauss and colleagues show that the Cyclin B1 protein is essential to make cells divide and that this protein must be constantly transported out of the nucleus to prevent cells dividing at the wrong time.

## Introduction

The eukaryotic cell cycle comprises the alternation between DNA replication and chromosome segregation and is driven by the Cyclin–Cdk family of protein kinases (Morgan, 2007). Most eukaryotic cells have multiple Cyclin–Cdks that are active at different stages of the cell cycle, but in essence, the control of the cell cycle can be reduced to an oscillation in Cyclin–Cdk activity: low activity allows the loading and firing of DNA replication origins, whereas high activity prevents origin loading and promotes mitosis (Noton and Diffley, 2000). In mammalian cells, mouse knockout studies have demonstrated that only a single Cdk, Cdk1, is essential for the cell cycle in the developing embryo (Santamaría et al., 2007), but it is not known which cyclins are essential to drive the cell cycle, particularly mitosis.

There are two classes of mitotic cyclins: the A- and the B-type cyclins. There are two closely related B-type cyclins in mammalian cells (a third Cyclin, named B3, is only distantly related by sequence and is restricted to germ cells; Nguyen et al., 2002). Cyclin B1 is essential for mouse development, because no homozygous Cyclin B1–null embryos are recovered at embryonic day 10 (Brandeis et al., 1998). In contrast, Cyclin B2 appears to be redundant in the mitotic cell cycle; the knockout mouse has no phenotype except a smaller litter size (Brandeis et al., 1998). The likely explanation for the primacy of Cyclin B1 is that whereas Cyclin B1 shuttles between the nucleus and the cytoplasm in interphase and binds to the mitotic apparatus in mitosis, Cyclin B2 is localized primarily to the Golgi apparatus (Jackman et al., 1995) and thus cannot phosphorylate the majority of substrates recognized by Cyclin B1. Indeed, the localization of the B-type cyclins is crucial to their function; in the absence of other B-type cyclins, Cyclin B2 can only affect the structure of the Golgi apparatus, whereas Cyclin B1 can reorganize the cytoskeleton and disassemble the nuclear lamina (Draviam et al., 2001). Similarly, frog Cyclin B can promote DNA replication when it is targeted into the nucleus by replacing its N terminus with that from Cyclin E (Moore et al., 2003).

Using a Cyclin B1–Cdk1–specific Förster resonance energy transfer (FRET) sensor, we found that human Cyclin B1–Cdk1 was first activated ∼20 min before nuclear envelope breakdown (NEBD), and upon activation, the complex immediately began to move into the nucleus (Gavet and Pines, 2010b). This redistribution to the nucleus has been suggested to be important to impose switch-like behavior on mitotic entry (Santos et al., 2012). Earlier studies had found that a constitutively nuclear Cyclin B1 did not perturb cell cycle timing, but combining this with a Cdk1 that was insensitive to Wee1 inhibition (Cdk1, T14A, and Y15F) caused premature mitotic entry (Jin et al., 1998).

Although Cyclin B1 is essential for mouse development, it is not known when it is first required. It may be essential for cell division from fertilization onward, like Cdk1 (Santamaría et al., 2007), or only later in development, like Cyclin A2 (Murphy et al., 1997). Until recently, the B-type cyclins were thought to be essential for mitosis in most organisms, except *Drosophila melanogaster*, where only Cyclin A is required (Lehner and O’Farrell, 1990; Jacobs et al., 1998). With the advent of RNAi, however, this notion has been challenged in mammalian cells. Several studies have reported that cells are still able to enter mitosis after depletion of Cyclin B1 (Bellanger et al., 2007; Gong et al., 2007; Soni et al., 2008; Gong and Ferrell, 2010). Thus, it has been proposed that, as in *Drosophila*, mammalian Cyclin B1 may be redundant with Cyclin A2 or with Cyclin A in cooperation with Cyclin B2 (Bellanger et al., 2007; Soni et al., 2008).

One important caveat to RNAi studies, however, is that they do not completely deplete a protein; therefore, with an enzyme such as a protein kinase, the small amount of protein that remains may provide sufficient activity to fulfill its function. A definitive answer to the question of whether Cyclin B1 is essential for cell division requires a true genetic null; therefore, we have analyzed the early cell cycles of Cyclin B1–null embryos using a mouse knockout line that was generated by Brandeis et al. (1998). In addition to defining the requirement for Cyclin B1 in mitosis, we have also explored the importance of the spatial control of Cyclin B1 in regulating mitosis. Here, we show that cells cannot divide without Cyclin B1; in the absence of Cyclin B1, cells arrest at the four-cell stage in G2 phase after DNA replication. Furthermore, we have developed a novel imaging approach to exploit this clean genetic background as a system in which we can analyze specific aspects of Cyclin B1 function by comparing the effects of injecting mRNA encoding wild type with mutant forms of Cyclin B1. These experiments demonstrate that the nuclear export of Cyclin B1 is crucial for the correct timing of mitotic entry, because constitutively nuclear Cyclin B1 promotes early entry to mitosis even when bound to endogenous Cdk1 that is sensitive to regulation by Wee1. In comparison, preventing Cyclin B1–Cdk1 from freely diffusing in the cytoplasm prevents its ability to promote mitosis.

## Results

### Embryos arrest at the four-cell stage without Cyclin B1

To determine the role of Cyclin B1 in cell division, we analyzed the early development of embryos from a Cyclin B1–knockout mouse line (Brandeis et al., 1998; gift of T. Hunt, Clare Hall Laboratories, London, England, UK). Brandeis et al. (1998) were unable to recover Cyclin B1^−/−^ embryos at embryonic day 10 but did not establish why embryos failed to develop to this stage. Thus, it was important to determine when the absence of Cyclin B1 prevented further development and whether this was because of an inability to divide or the consequence of compromised divisions or some other function of Cyclin B1 that became important between fertilization and day 10.

Cyclin B1^+/−^ heterozygotes are fully viable; therefore, we set up crosses to obtain embryos with +/+, +/−, and −/− (null) Cyclin B1 genotypes for analysis. To determine when the Cyclin B1^−/−^ embryos stopped developing, we cultured zygotes in vitro for 3.5 d up to the blastocyst stage and, in parallel, used time-lapse imaging of embryos to characterize the mitotic divisions. We did not have a live-cell marker for the deletion; therefore, we had to determine the genotype of the embryos at the end of the experiment using a PCR protocol specifically for low and variable amounts of DNA (see Fig. S1 A and Materials and methods). To facilitate analysis in live-imaging experiments, we developed an imaging device, which we called the Multiwell Immobilization and Imaging, or MI^2^, Chip (Dolomite Microfluidics). Using this device, we could culture and image large numbers of embryos at high resolution for several days and recover each embryo for genotyping at the end of the experiment (Materials and methods and Fig. S1 B).

**Figure 1.**
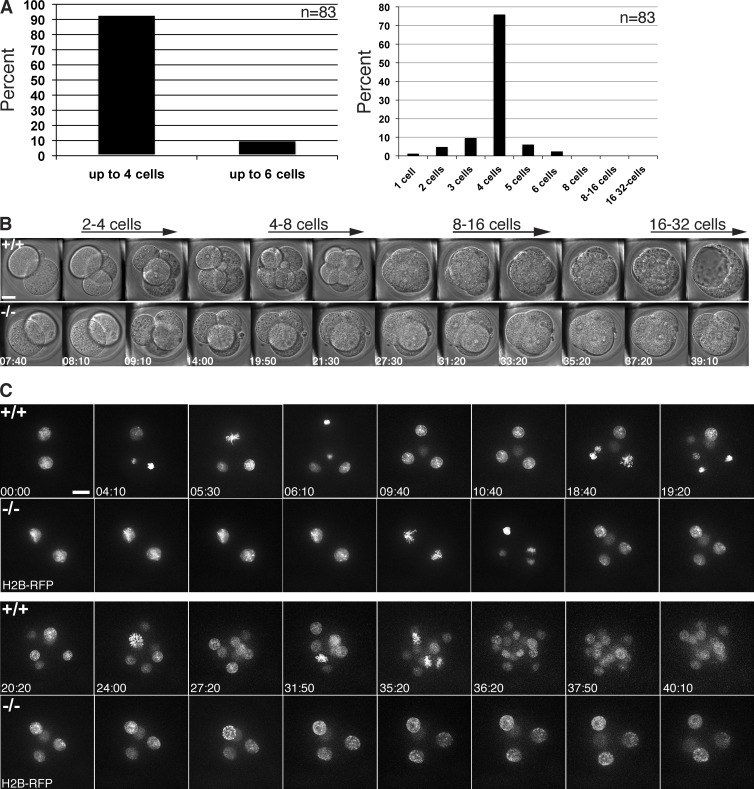
**Cyclin B1^−/−^ embryos arrest at the four-cell stage after two normal divisions.** (A) Zygotes obtained from crosses of viable Cyclin B1^+/−^ mice were cultured up to 3.5 d in vitro (left). Out of 83 Cyclin B1 homozygous–null embryos, 92% arrest at the four-cell stage or earlier (23 independent experiments). Distribution of the number of blastomeres found in Cyclin B1^−/−^ embryos (right); 75% arrest at the four-cell stage; in 6% of embryos, one (or in 2%, two) blastomeres can divide once more. (B) Differential interference contrast time-lapse imaging of a wild-type (top) and a Cyclin B1^−/−^ embryo (bottom) from the same litter (bar, 20 µm; time in hours:minutes). Representative frames from Video 1 shown from the late two-cell stage till the beginning of the 32-cell stage and blastocoel formation. No difference in the temporal dynamics of the first two divisions was observed between wild-type and knockout embryos, but the Cyclin B1^−/−^ embryo arrests after the second division. (C) H2B-RFP mRNA was injected into zygotes to visualize DNA in wild-type (top) and Cyclin B1^−/−^ embryos (bottom). Representative frames from Video 2, shown from the late two-cell stage until the blastocyst stage. Chromosome condensation, metaphase plate formation, and chromosome segregation were indistinguishable between wild-type and Cyclin B1^−/−^ embryos. In 80% of cases (five of six embryos in three independent experiments), arrested Cyclin B1^−/−^ embryos showed variable and transient rounds of incomplete chromatin condensation in one or more blastomeres (see time point 27 h:20 min, bottom, and Video 2). Bar, 20 µm. Time in hours:minutes.

After 3.5 d of culture, we found that the great majority (92%, *n* = 83) of Cyclin B1–null embryos had arrested before or at the four-cell stage (Fig. 1 A, left). 75% of Cyclin B1–null embryos arrested exactly at the four-cell stage, but in rare cases, one (6%) or two (2%) of the blastomeres at the four-cell stage underwent one additional division (Fig. 1 A right). Thus, Cyclin B1–null embryos had the earliest arrest phenotype of any Cyclin knockout analyzed to date.

### Cyclin B1–null embryos undergo two normal divisions

To determine why embryos stopped dividing in the absence of Cyclin B1, we compared the development of wild-type and Cyclin B1–null embryos by high-resolution time-lapse imaging and found their divisions to be indistinguishable in timing and morphology up to the four-cell stage (Fig. 1 B and Video 1). (It should be noted that blastomere divisions are not synchronous in the mouse embryo, and the onset of division at any given round of division can vary up to several hours between embryos of the same litter; Bischoff et al., 2008.) After the second division, however, Cyclin B1–null embryos stopped dividing, whereas the wild-type and heterozygote embryos continued to develop up to the blastocyst stage (Video 1). Although the Cyclin B1–null embryos arrested, they showed no obvious signs of deterioration or apoptosis. Moreover, we noted that the Cyclin B1^−/−^ embryos appeared to undergo compaction at approximately the same time as wild-type embryos (when the latter reached the mid-to-late eight-cell stage). Thus, consistent with earlier studies (Day et al., 1998; Goval and Alexandre, 2000), the timing mechanism for compaction appeared to be independent of cell division in the mouse embryo.

To analyze cell division in Cyclin B1^−/−^ embryos in greater detail, we injected zygotes with synthetic mRNA encoding histone H2B-RFP to visualize the chromosomes. Time-lapse imaging showed that chromosome condensation, alignment, and segregation were indistinguishable between wild-type and Cyclin B1^−/−^ embryos during the first two divisions (Video 2 and Fig. 1 C). Once the Cyclin B1^−/−^ embryos had arrested after the second division, however, it was noticeable that in 80% of embryos, the chromatin underwent partial condensation and decondensation to varying degrees in one or more of the blastomeres (Fig. 1 C and Video 2). These condensations, however, did not progress to the pattern of chromosome condensation in late prophase wild-type cells, and the nuclear envelope never broke down. These partial chromosome condensation events might have been caused by residual maternal Cyclin B1 RNA or possibly by rising levels of Cyclin A2–Cdk activity. Similarly, differences in the degree of chromosome condensation between blastomeres might have been caused by variable levels of residual Cyclin B1 message. To address this, we measured Cyclin B1 mRNA levels in four-cell–arrested embryos using quantitative single-embryo RT-PCR. We measured LacZ transcript levels as a marker for Cyclin B1^+/−^ and Cyclin B1^−/−^ embryos and found that there was a bimodal distribution of Cyclin B1 levels in LacZ-positive embryos (Table S1); Cyclin B1 mRNA was either not detectable above background or present at 100- to 1,000-fold greater levels. Because the DNase1 treatment required for RT-PCR precludes direct genotyping, we interpret this as the difference between the Cyclin B1^−/−^ and the Cyclin B1^+/−^ embryos.

### Cyclin B1–null embryos cannot complete entry to mitosis even when antagonistic phosphatases are inhibited

For cells to enter mitosis, it is not sufficient simply to activate Cyclin B1–Cdk1; the phosphatases that antagonize Cyclin B–Cdk1 must also be inhibited to prevent a futile cycle, and this is achieved by an inhibitor generated by the Greatwall kinase (Castilho et al., 2009; Gharbi-Ayachi et al., 2010; Mochida et al., 2010). This raised the possibility that another Cyclin–Cdk might be able to drive cells into mitosis in the absence of Cyclin B1 if we inhibited PP2A; indeed, inhibiting protein phosphatases with okadaic acid (OA) can overcome the cell cycle arrest in mouse spermatocytes lacking Cyclin A1 and drive them into meiosis (Liu et al., 2000). As OA has been used previously in mammalian cells and mouse oocytes to inhibit PP2A (Chang et al., 2011), we used arrested mouse oocytes as an assay system and established that 250 nM OA induced NEBD (germinal vesicle breakdown; Fig. S2A). Therefore, we treated Cyclin B1–null embryos arrested at the four-cell stage with 250 nM OA and assayed the effect by time-lapse microscopy. After 20 h, 80% (*n* = 15) of Cyclin B1–null embryos remained arrested with uncondensed or only partially condensed chromatin. Only in 20% of embryos, which could have had a higher residual level of Cyclin B1 maternal mRNA, did the chromatin condense and disperse (Fig. S2 B; and Video 3, middle). To analyze the phenotypes further, we coinjected Cyclin B1–null embryos with EB3-GFP and H2B-RFP. Fig. S2 (C and D) and Videos 4 and 5 show that the nuclear envelope did break down in the majority of cells, indicated by the entry of tubulin into the nuclear region, but cells made no attempt to organize a spindle. In the minority of cells where condensed DNA dispersed, disorganized microtubule bundles formed transiently but without organizing into a proper spindle structure. Thus, we conclude that Cyclin B1 is required to complete mitotic entry even in the absence of antagonistic PP2A phosphatase activity.

### Cyclin B2 does not make an apparent contribution to early cell divisions

Our results showed that mouse Cyclin B1 is essential in the earliest cell divisions and that from the four-cell stage onward, its function cannot be replaced by Cyclin A2 or B2. Brandeis et al. (1998) previously showed that Cyclin B2 was not essential for development, but later siRNA studies in cell culture concluded that Cyclin B2 could compensate for Cyclin B1 in mitosis (Bellanger et al., 2007; Soni et al., 2008). It was important, therefore, to assess whether Cyclin B2 made any contribution to driving the second division in Cyclin B1^−/−^ embryos.

We crossed Cyclin B2^−/−^ mice (a gift of T. Hunt) with Cyclin B1^+/−^ mice and analyzed the development of the Cyclin B1^−/−^/Cyclin B2^−/−^ embryos. If Cyclin B2 contributes effectively to the first two divisions, then one would expect that these embryos would either arrest at an earlier stage or exhibit more compromised second divisions. We found, however, that the Cyclin B1/B2 double-knockout embryos showed the same arrest phenotype as the Cyclin B1–knockout embryos at the four-cell stage (Fig. S3). Thus, it appears that in the embryo Cyclin B2 does not compensate for Cyclin B1 in promoting mitosis.

### Cyclin B1–null embryos arrest after completing S phase

Our imaging data showed that Cyclin B1^−/−^ embryos exited mitosis normally at the second division. Therefore, we wished to establish at which stage cells arrested in the next cell cycle in the absence of Cyclin B1. To achieve this, we used proliferating cell nuclear antigen (PCNA) as a marker to analyze S phase entry and progression. PCNA, an essential component of the replication machinery, localizes to replication foci, and PCNA foci can be used as a marker for cells in S phase (Leonhardt et al., 2000); the size and distribution pattern of foci can even be used to distinguish between early, mid-, and late S phase cells.

**Figure 2.**
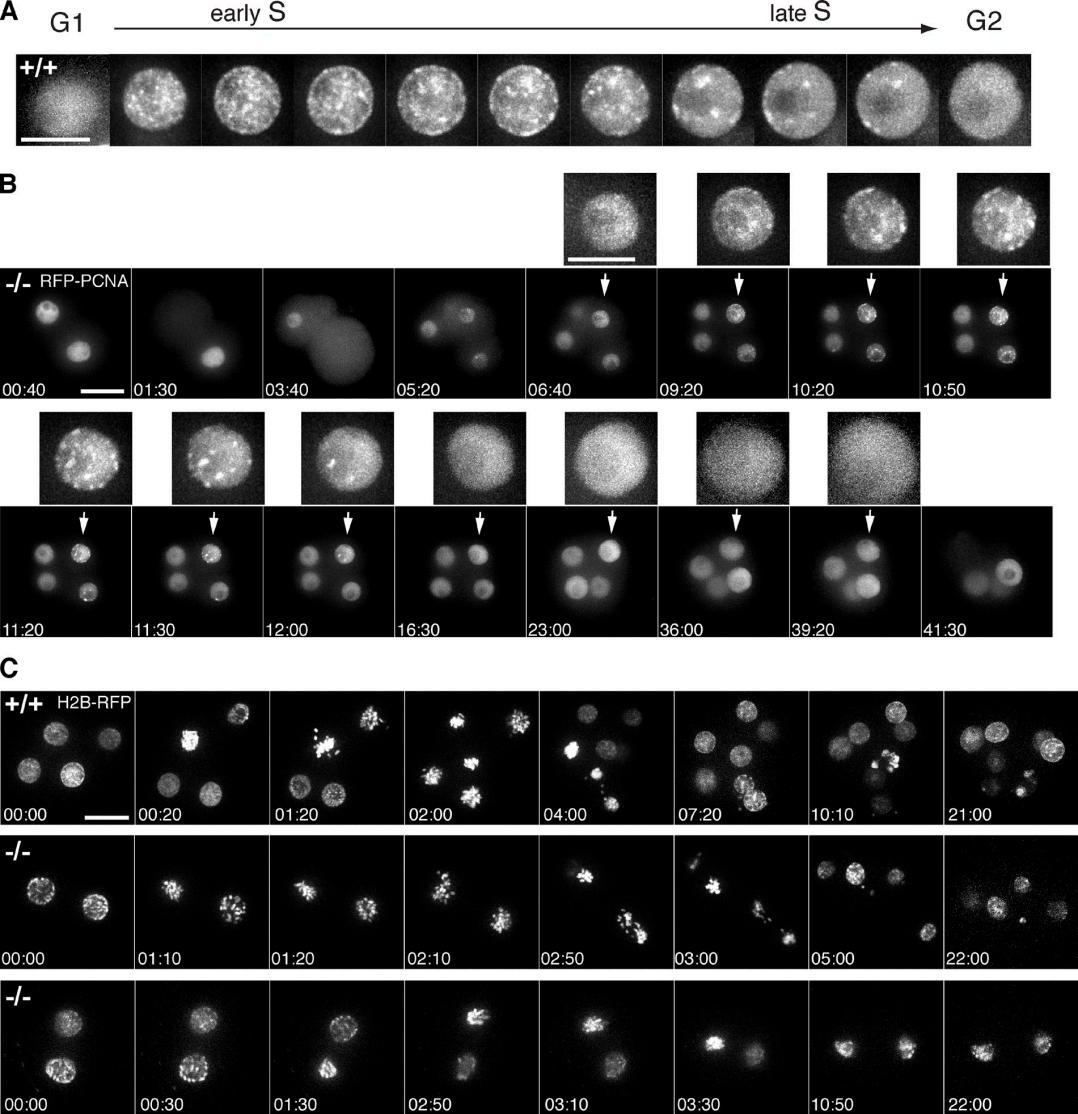
**Cyclin B1^−/−^ embryos arrest in G2 phase after completing S phase.** (A) Progress through S phase in the early mouse embryo can be monitored by injection of RFP-PCNA mRNA. The characteristic patterns of PCNA foci during S phase, and its homogenous distribution in G1 and G2 phases, were used to analyze the timing of different cell cycle phases in the early embryo. Bar, 10 µm. (B) Zygotes were injected with RFP-PCNA and representative frames of the Cyclin B1^−/−^ embryo in Video 9 are shown from the two-cell stage until the corresponding wild-type reaches the blastocyst stage (bar, 20 µm). Enlarged inserts on top shows how one nucleus progresses normally through S phase after the second division and then arrests with homogenous PCNA distribution indicative of G2 phase (*n* = 5 embryos in three independent experiments). Bar, 10 µm (insets). (C) Representative frames of the RFP channel in Video 7. One blastomere was injected with H2B-RFP mRNA at the two-cell stage. Wee1 inhibitor was added to arrested Cyclin B1–null embryos at the time when wild-type embryos had started the four- to eight-cell division. Inhibitor was added during first time point. Wild-type (top) and Cyclin B1^−/−^ (middle) chromatin condensation and some dispersal indicating NEBD (1 of 13 embryos); Cyclin B1^−/−^ (bottom): chromatin condensation but no dispersal. Bar, 20 µm; time in hours:minutes.

To our knowledge PCNA had not previously been used to study S phase in live early mouse embryos. Therefore, we first had to establish the normal pattern of foci distribution. We microinjected embryos with synthetic mRNA encoding RFP-PCNA and analyzed the fluorescence pattern by high-resolution live-cell imaging. This showed that early mouse embryos exhibited the characteristic progression of foci patterns reported in cultured mammalian cells (Leonhardt et al., 2000): the sudden appearance of many evenly distributed, small foci marked S phase onset, followed by reorganization from mid- through late S phase into progressively fewer, bigger foci mainly localized around nucleoli and at the nuclear periphery, until all foci disappeared at the end of S phase (Fig. 2 A). In G1 and G2 phases, the PCNA signal showed homogenous nuclear distribution, and the nuclear signal was lost at NEBD and reappeared when the nuclear membrane reformed in telophase.

To determine the exact cell cycle stage when cells arrested in the absence of Cyclin B1, we injected RFP-PCNA at the zygote stage and followed development by time-lapse fluorescence imaging. After the second division, the nuclei of wild-type and Cyclin B1^−/−^ embryos passed through a very short G1 phase of ∼20 min and began to form small replication foci that subsequently developed into progressively bigger and fewer foci, indicating that the nuclei were proceeding through S phase (Fig. 2 B and Video 6). Comparing wild-type and Cyclin B1–null embryos showed that there was no apparent difference in the timing of DNA replication or patterns of foci, but once the last focus had disappeared, marking the end of S phase, the nuclei of the Cyclin B1–null embryos arrested with the homogenous nuclear distribution of PCNA characteristic of G2 phase.

### Cyclin B1–null embryos are not arrested by a Wee1-dependent checkpoint

It was possible that the Cyclin B1–null embryos arrested in G2 phase as a consequence of activating a checkpoint because of incomplete DNA replication or DNA damage rather than the lack of Cyclin B1. To test this, we inhibited the Wee1 kinase, which is required for the unreplicated and DNA damage checkpoints that prevent mitotic entry by phosphorylating Cyclin B1–Cdk1 (Russell and Nurse, 1987; Enoch and Nurse, 1990; O’Connell et al., 1997; van Vugt et al., 2004). Adding the Wee1 inhibitor MK1775 (Hirai et al., 2009) dramatically shortened G2 phase to ∼30 min (31 ± 16 min [mean ± SD]) in wild-type and heterozygous embryos (*n* = 23, measured at the 8-cell to 16-cell transition in five independent experiments), which is only 25% of the expected length of G2 phase (for control G2 timing, see Fig. 6, A and B). Despite this effect, adding the inhibitor to four-cell Cyclin B1^−/−^ embryos only led to the initiation of prophase events as seen with OA; it did not induce normal chromosome condensation, alignment, or metaphase plate formation in 12 out of 13 embryos (four independent experiments), although in some blastomeres, the chromosomes did partially condense (Fig. 2 C and Video 7). Thus, Cyclin B1^−/−^ embryos are not held in G2 phase by a Wee1-dependent checkpoint.

### Cyclin B1 is essential for mitosis

**Figure 3.**
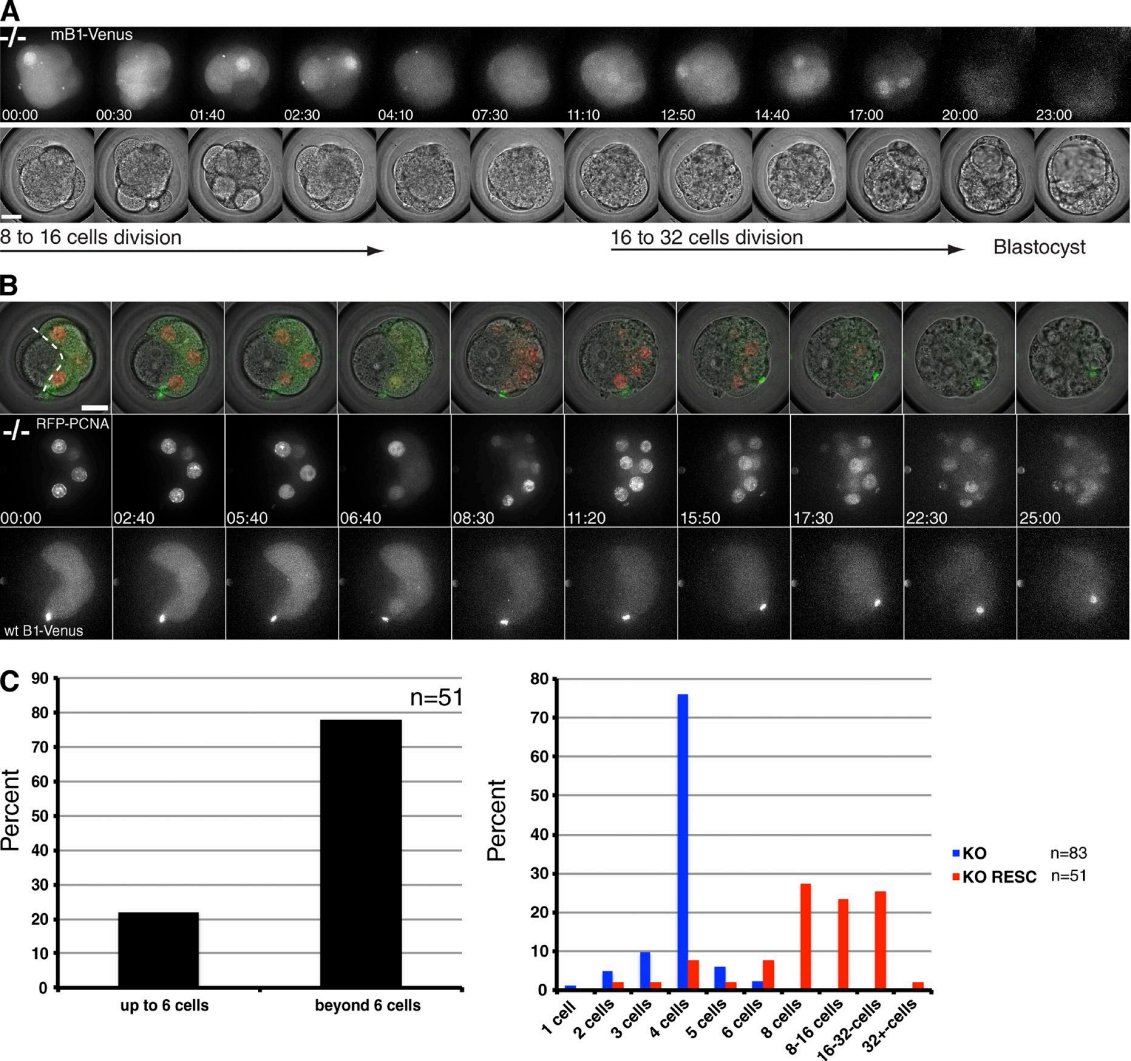
**Cyclin B1–null embryos can be driven through up to three divisions by injecting wild-type Cyclin B1 mRNA.** (A) Wild-type mouse Cyclin B1–Venus mRNA (mB1-Venus) was injected into both blastomeres at the two-cell stage. Representative frames of Video 8 are shown, beginning at the eight-cell stage; wild-type Cyclin B1–Venus (top) and transmitted light channels (bottom). Note the clearly visible nuclear import of Cyclin B1 before NEBD. Bar, 20 µm. (B) Additional markers such as PCNA were coinjected with wild-type or mutant Cyclin B1 mRNA in one blastomere at the two-cell stage. Representative frames of Video 9 are shown, from the eight-cell stage onward. The two uninjected blastomeres, left of the white dotted line, serve as an internal control and remain arrested whereas the right half of the embryo progresses up to the 16-/32-cell stage. Top row shows channel merge of single plane through the center of the embryo; middle row shows projection of the RFP-PCNA channel. Bottom row shows projection of mB1-Venus channel (bar, 20 µm). (C) Quantification of rescue of Cyclin B1^−/−^ embryos by injection of wild-type mouse Cyclin B1 mRNA; 78% of Cyclin B1^−/−^ embryos divide beyond the arrest stage (taken as the six-cell stage to include the rare cases in which one or two blastomeres divide beyond the four-cell stage; *n* = 51, 10 independent experiments; left). Variation in the ability of injected wt mRNA to rescue (red bars, *n* = 51) compared with nonrescued Cyclin B1^−/−^ embryos (blue bars, *n* = 83, 21 independent experiments; right); ∼25% of embryos respectively divided to the 8-cell stage, to the 16-cell stage, and up to the 32-cell stage.

All our results indicated that Cyclin B1 was essential for cells to begin mitosis. To test this and to establish whether Cyclin B1–null embryos could be used to study aspects of Cyclin B1 function and regulation, we performed “rescue” experiments by injecting synthetic mRNA into zygotes, or into one blastomere at the two-cell stage. Injecting synthetic mRNA encoding Cyclin B1 drove Cyclin B1–null embryos through up to three further divisions to the 32-cell stage and blastocyst formation (Fig. 3 A and Video 8). We measured the amount of Cyclin B1–Venus protein translated from injected mRNA and compared this to endogenous levels by immunoblotting (Fig. S4 A). We found that Cyclin B1–Venus was expressed at levels very similar to the amount of endogenous Cyclin B1 protein in wild-type embryos and that endogenous Cyclin B1 protein was itself suppressed in these injected embryos (Fig. S4 C).

To analyze more accurately the ability of Cyclin B1 to drive cell division, we coinjected wild-type Cyclin B1 mRNA with mRNA encoding fluorescent markers of chromatin (histone H2B), microtubules (β-tubulin; not depicted), or replication foci (PCNA; Fig. 3 B and Video 9). We injected the synthetic mRNAs into only one blastomere of a two-cell Cyclin B1^−/−^ embryo to provide us with an internal control; the uninjected blastomere usually divided only once and then arrested, whereas the injected blastomeres continued to divide up to three more times. In total, 78% (*n* = 51) of injected embryos underwent at least one complete division beyond the four-cell arrest stage (Fig. 3 C, left); ∼25% of these divided twice and 25% three times (Fig. 3 C, right). Thus, we conclude that the G2 phase arrest of Cyclin B1^−/−^ cells can be rescued by injection of Cyclin B1 mRNA and that Cyclin B1 is essential for cells to enter mitosis.

### Small amounts of Cyclin B1 are sufficient to trigger mitosis

**Figure 4.**
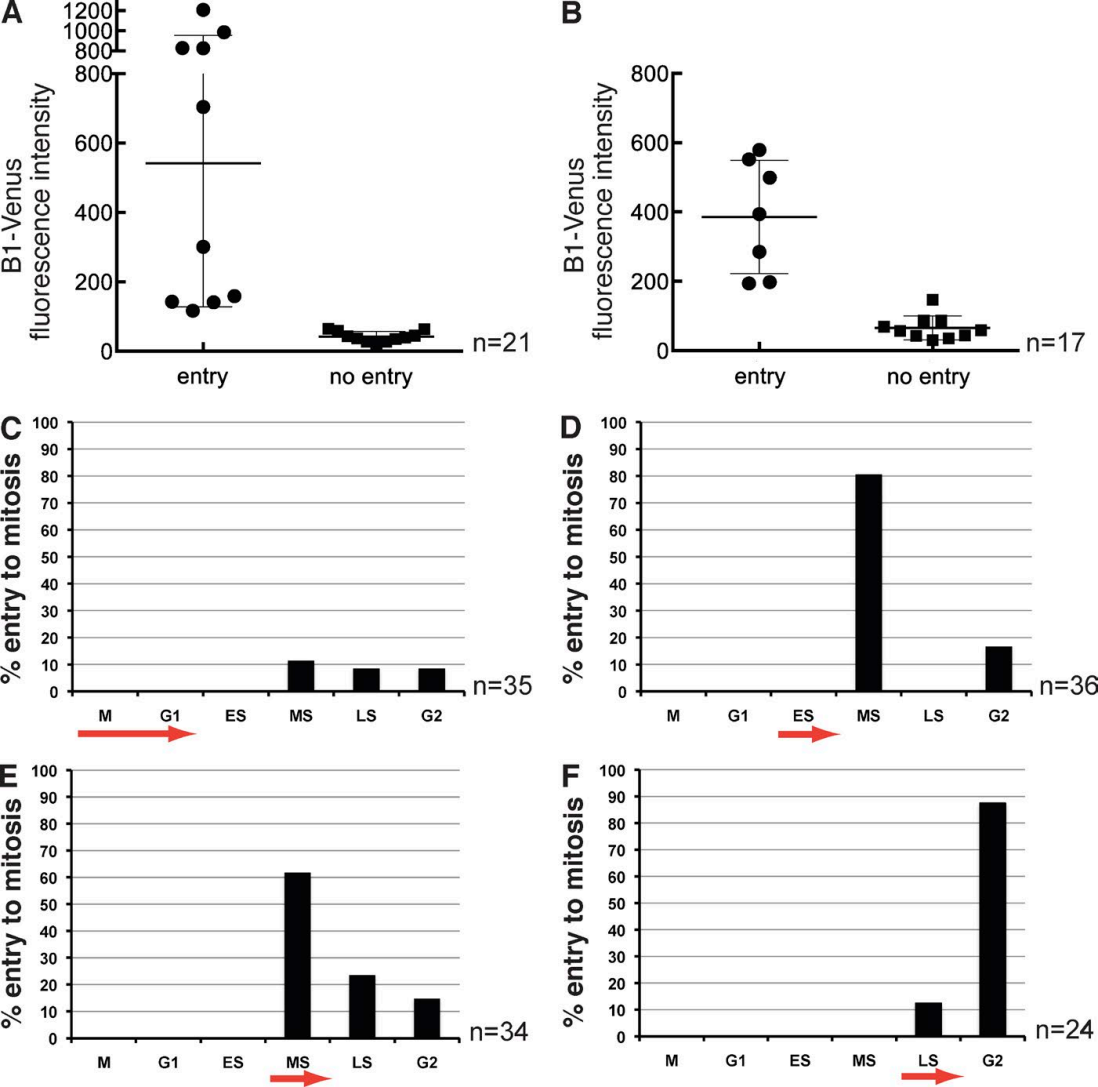
**Low amounts of Cyclin B1 are sufficient to trigger mitosis and the threshold is reached by mid S phase.** (A) To establish the threshold of Cyclin B1–Venus that can drive null cells into mitosis, fluorescence intensity was used as a read-out for the amount of protein and measured in individual blastomeres of −/− embryos; y axis arbitrary intensity units. The threshold concentration to trigger mitosis lies between 100 and 200 units above background intensity, corresponding to ∼1/10 of the sampled intensity range (*n* = 21 blastomeres from five experiments). (B) Wild-type Cyclin B1–Venus was injected into one blastomere of −/− embryos at the two-cell stage to rescue the arrest phenotype. Signal intensity was measured at the 8- to 16-cell transition after addition of Wee1 inhibitor. Only injected cells with a Cyclin B1 level above a threshold of ∼150 arbitrary units can be forced into mitosis by Wee1 inhibition; *n* = 17, 1 experiment. Error bars are the SD of the mean. (C–F) To test whether there is a time dependency of the inhibitory phosphorylation of Cdk1 by Wee1 the inhibitor MK1775 was added at different phases of the cell cycle to +/+ and +/− embryos. (C) Inhibitor added during mitosis or G1. Only 30% of cells enter to mitosis. 70% of cells arrest after a variable S phase (*n* = 35, six experiments). (D) Inhibitor added during early S phase leads to mitotic entry during mid-S phase in most cases; 80% (*n* = 36; six experiments). (E) Inhibitor added during mid-S phase forces cells into mitosis mostly in mid- and late S (together 85%; *n* = 34, six experiments). (F) Inhibitor added during late S phase drives cells into mitosis mostly with a much shortened G2 (see text for details; *n* = 24, five experiments). ES, early S phase; LS, late S phase; MS, mid-S phase.

The question now arose of how to reconcile our results with those from siRNA experiments in tissue culture cells, where reducing Cyclin B1 to low levels had little apparent effect on the ability of cells to enter mitosis. One possibility is that only small amounts of Cyclin B1 are required for mitosis, which can be tested reliably only in a true null background. To pursue this, we tested whether there was a critical threshold of Cyclin B1 needed to trigger mitosis in the Cyclin B1^−/−^ embryos. We injected different concentrations of synthetic mRNA encoding Cyclin B1–YFP into embryos and correlated the amount of Cyclin B1–YFP fluorescence with the ability to trigger mitosis. We found that a relatively clear but small threshold amount of Cyclin B1 was sufficient to trigger mitotic entry (Fig. 4 A).

We considered the possibility that the amount of Cyclin B1 necessary to trigger mitosis might be reduced further in cells in which we inhibited Wee1, but we found that a similar threshold value of Cyclin B1 had to be reached in cells treated with the MK1775 inhibitor (Fig. 4 B). Treating wild-type embryos at different stages of the cell cycle with MK1775 showed that embryos from mid-S phase onward were able to enter mitosis, potentially indicating that the threshold of Cyclin B1 required for mitosis was reached in mid-S phase (Fig. 4, C–F).

### Nuclear Cyclin B1 can drive mitosis in Cyclin B1^−/−^ embryos

Our results revealed the potential to use Cyclin B1–null embryos as an experimental system to study Cyclin B1 function and regulation by injecting mRNAs encoding mutant forms of Cyclin B1 into a genetically null background. Moreover, the large cell size of the early mouse embryo makes this system particularly well suited to study spatial controls using a 3D imaging approach.

One of the most prominent characteristics of Cyclin B1 is its dynamic localization throughout the cell cycle. We and others have shown that Cyclin B1 shuttles between the cytoplasm and the nucleus, but its greater rate of export over import ensures that the majority of Cyclin B1 is cytoplasmic throughout interphase (Hagting et al., 1998; Toyoshima et al., 1998; Yang et al., 1998). At mitosis, cytoplasmic Cyclin B1 is rapidly imported into the nucleus concomitant with the activation of its partner kinase, Cdk1 (Gavet and Pines, 2010a,b). Although we understand the mechanism by which Cyclin B1 is maintained in the cytoplasm, the function of this spatial distribution is still not understood. It has been variously suggested that the nuclear export of Cyclin B1 is required to prevent its S phase–promoting activity (Moore et al., 2003), or in parallel with phosphorylation on T14 and Y15 of Cdk1 to be part of the mechanism to prevent entry to mitosis (Santos et al., 2012), in particular after DNA damage (Jin et al., 1998), or to act as a spatial positive feedback loop to impose switch-like behavior on the entry to mitosis (Santos et al., 2012).

**Figure 5.**
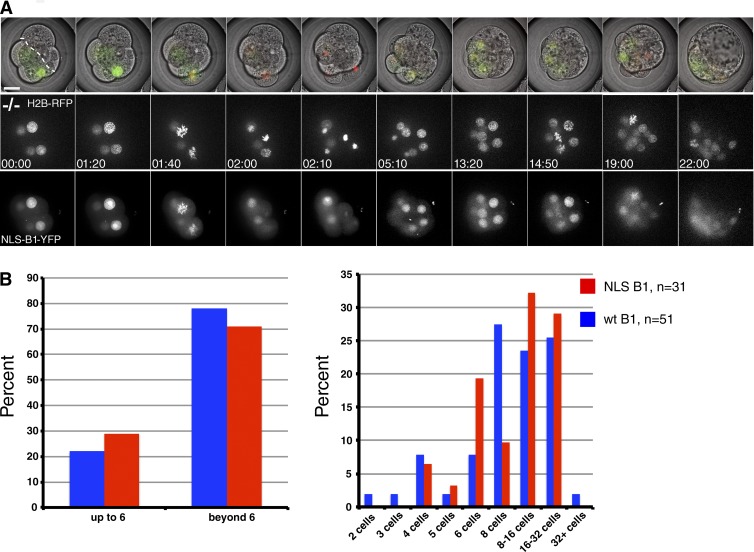
**Nuclear Cyclin B1 can drive mitosis in Cyclin B1^−/−^ embryos.** (A) Frames of Video 10 are shown from the eight-cell stage onwards. Synthetic mRNAs encoding nuclear Cyclin B1 and H2B-RFP were injected in one blastomere at the two-cell stage. The uninjected half of the embryo is to the right of the dotted line, the injected half of the embryo divides up to the 16-/32-cell transition. Top row shows the channel merge of a single central plane through the embryo. Middle row shows projection of the H2B-RFP channel. Bottom row shows projection of the nuclear Cyclin B1 channel. (B) Comparison of rescue efficiency of wild-type (wt; blue, *n* = 51) and nuclear Cyclin B1 mRNA (red, *n* = 31, eight independent experiments) in driving Cyclin B1^−/−^ embryos beyond the arrest stage (left). Comparison of the number of divisions that wild-type and nuclear Cyclin B1 can promote in Cyclin B1^−/−^ embryos (right). Bar, 20 µm.

Previous studies on the role of Cyclin B1 localization in the cell cycle had been complicated by the presence of endogenous Cyclin B1, but our genetic null system overcame this issue. Therefore, we injected synthetic mRNA encoding Cyclin B1 with a F146A substitution to inactivate its nuclear export sequence (Hagting et al., 1998) plus a 7–amino acid nuclear localization sequence (NLS) to ensure that Cyclin B1 remained in the nucleus throughout the cell cycle. This mutant is hereafter called nuclear Cyclin B1. To establish the effect on the cell cycle of retaining Cyclin B1 in the nucleus, we injected mRNA encoding wild-type or nuclear Cyclin B1 into one blastomere at the two-cell stage. In some embryos, H2B-RFP was coinjected to assess chromosome segregation and mitotic progression (Fig. 5 A and Video 10). These experiments showed that wild-type (cytoplasmic) and nuclear Cyclin B1 had very similar abilities to drive successful rounds of cell divisions (Fig. 5 B). Thus, at least in early embryonic divisions, it appears that the continual nuclear export of Cyclin B1 is not required for cells to progress through the cell cycle.

### Nuclear Cyclin B1 accelerates entry into mitosis

**Figure 6.**
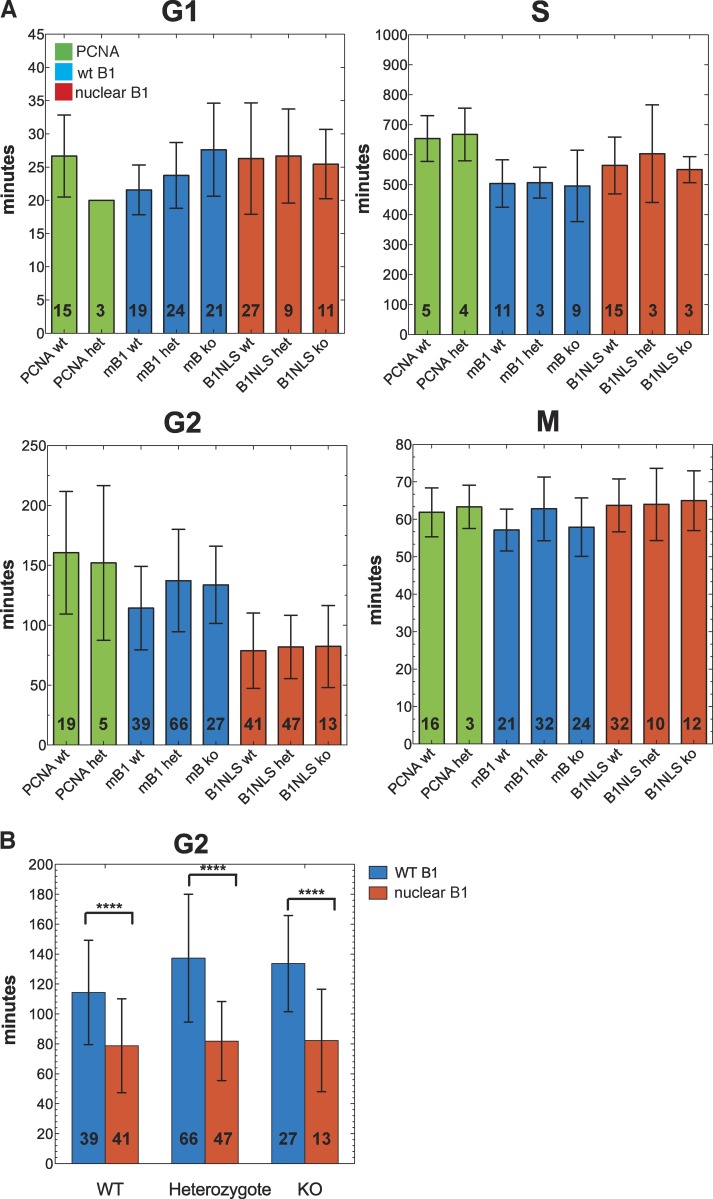
**Nuclear Cyclin B1 accelerates entry into mitosis.** (A) The lengths of different phases of the cell cycle were quantified after injecting either PCNA alone (PCNA, green; three independent experiments), wild-type Cyclin B1 (mB1, blue; four independent experiments), or nuclear Cyclin B1 (B1NLS, red; five independent experiments). y axis shows time in minutes (mean; error bars represent ±SD of the mean) is shown for each of the genotypes: +/+ (wt), +/− (het), and −/− (ko). Numbers inside bars indicate number of divisions analyzed. (B) The greatest difference in timing between wild-type and nuclear Cyclin B1 was in G2 phase where nuclear Cyclin B1 reduced G2 length by ∼40% (P < 0.0001, two-sided *t* test; error bars: ±SD of the mean; ****, P < 0.0001).

Although both cytoplasmic and nuclear Cyclin B1 mRNAs could rescue up to three complete cell cycles, we wanted to determine whether the localization of Cyclin B1 would affect cell cycle timing. Therefore, we coinjected mRNA encoding RFP-PCNA as a marker to measure the lengths of the different cell cycle stages and analyzed the timing at the 8- to 16-cell division. We defined S phase as beginning with the appearance of PCNA foci and ending with their disappearance. The subsequent period with a homogeneous nuclear PCNA signal was classified as G2 phase. We defined the onset of mitosis as the time point when the homogeneous PCNA nuclear signal was dispersed at NEBD and G1 phase as the time between the reappearance of the PCNA nuclear signal (after the nuclear envelope reformed) until the first appearance of replication foci. To our knowledge, this is the first attempt to measure the lengths of specific phases of the cell cycle in live preimplantation mouse embryos. The cell cycle timings that we measured (Fig. 6) agreed well with previous more indirect measures of cell cycle phase lengths in the early mouse embryo (Streffer et al., 1980).

In contrast to studies transfecting Cyclin B1 and Cdk1AF into human cells (Jin et al., 1998), our experiments showed that constitutively nuclear Cyclin B1 had little effect on the duration of mitosis (Fig. 6 A), nor did it appear to have any measurable effect on DNA replication, because both G1 and S phase durations were the same as in embryos injected with wild-type Cyclin B1. Thus, unlike in *Xenopus laevis* embryos (Moore et al., 2003), it appears that mammalian Cyclin B1 does not have to be exported from the nucleus to prevent it interfering with DNA replication. Nuclear Cyclin B1 did, however, significantly accelerate entry to mitosis after the end of DNA replication, because G2 phase was 40% shorter than normal (Fig. 6 B), even though it had only endogenous Cdk1 to bind. Thus, in addition to Wee1/Myt1– and Cdc25-mediated regulation of Cdk1, Cyclin B1 must be exported into the cytoplasm for cells to regulate the correct timing of mitosis.

### Tethering Cyclin B1–Cdk1 to the plasma membrane prevents mitotic entry

As nuclear Cyclin B1 can trigger normal mitosis, and because its forced nuclear localization affects only the timing of mitotic entry, our results also indicate that nuclear Cyclin B1 alone can sufficiently activate Cdk1 and the relevant feedback loops required for mitotic entry. However, the importance of cytoplasmic spatial control to initiate early mitotic events before NEBD is still not well understood. This is difficult to study in systems without a clean genetic null background; therefore, we asked to what extent the nuclear–cytoplasmic localization of Cyclin B1 is essential to trigger the first events of mitotic entry by holding Cyclin B1 in the cytoplasm. To achieve this, we used a C-terminal Caax domain (Cyclin B1–RFP–Caax) to retain Cyclin B1 at the plasma membrane.

**Figure 7.**
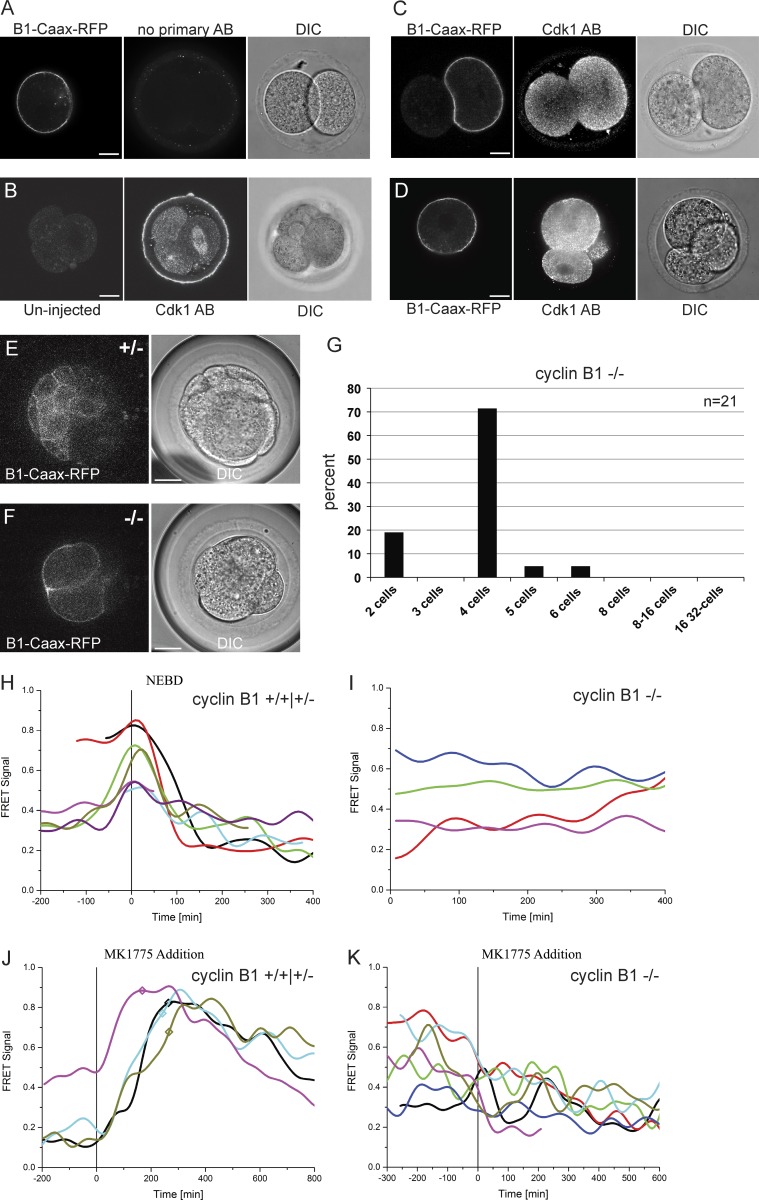
**Tethering Cyclin B1–Cdk1 to the plasma membrane perturbs its activation.** (A–D) Cdk1 antibody staining in Cyclin B1–RFP–Caax mRNA-injected embryos. (A) No primary control. (B) Uninjected embryo showing correct localization of Cdk1 on the spindle during mitosis (right blastomere) and throughout the cell in interphase (left two blastomeres). (C and D) Representative example sections showing Cdk1 localization in embryos injected with Cyclin B1–RFP–Caax. To test the ability of membrane-bound Cyclin B1 to rescue Cyclin B1^−/−^ embryos, synthetic mRNA encoding wild-type Cyclin B1–RFP–Caax was injected into one blastomere at the two-cell stage. Representative sections of live embryos at the 32-/64-cell stage for heterozygote (E) and knockout (F) genotypes show localization of membrane targeted cyclin B1. (G) Cell numbers in the injected and uninjected side were counted at this stage and the great majority (90%) of injected embryos arrested either at the four-cell stage or before (*n* = 21, 12 experiments) demonstrating the inability of membrane-bound cyclin B1 to rescue B1^−/−^ embryos. (H–K) Embryos injected with Cyclin B1–RFP–Caax mRNA were coinjected with a Cdk1 FRET sensor to show changes in levels of Cdk1 phosphorylation. Although Cyclin B1^+/+^ and Cyclin B1^+/−^ embryos show a normal increase of Cdk1 activation before NEBD and a subsequent decrease (H), Cyclin B1^−/−^ embryos remained at randomly fluctuating levels (I). (J and K) To test whether Wee1 inhibition can trigger Cdk1 activation in Cyclin B^−/−^ embryos with membrane-tethered Cyclin B1, FRET imaging was performed to detect changes in Cdk1 phosphorylation. Representative FRET signal graphs aligned to the time point of Wee1 inhibitor (MK 1775) addition. (J) Cyclin B1^+/+^ and Cyclin B1^+/−^ embryos show a clear response after addition of MK1775 (NEBD marked by squares on the graph). (K) Cyclin B1^−/−^ embryos injected with membrane-tethered Cyclin B1 showed no response in Cdk1 phosphorylation. There was no difference in FRET measurements close to or away from the membrane. Bars, 20 µm. Imaging interval in FRET analysis, 8 min.

We first checked that membrane-tethered Cyclin B1 could bind to Cdk1 by staining embryos with anti-Cdk1 antibodies and found that expressing Cyclin B1–RFP–Caax caused some of the Cdk1 to relocalize to the plasma membrane (Fig. 7, A–D). Thus, tethering Cyclin B1 to the plasma membrane does not preclude it binding to Cdk1. (Note that our quantification of Cyclin B1 and Cdk1 levels in embryos showed that Cdk1 is in fourfold excess to Cyclin B1; therefore, only a minority of Cdk1 would be expected to relocalize to the membrane; Fig. S4, A and C.) Next we tested whether Cyclin B1–Caax could rescue Cyclin B1^−/−^ embryos by injecting synthetic mRNA encoding Cyclin B1–RFP–Caax. As shown in Fig. 7 (E–G), wild-type and heterozygous embryos developed normally to the 32-cell stage, whereas Cyclin B1^−/−^ embryos arrested in 90% of cases at the four-cell stage or earlier (Fig. 7, F and G), the normal time point for Cyclin B1^−/−^ cells to arrest. The inability of membrane-bound Cyclin B1–Cdk1 to drive mitosis could have different explanations, the most obvious of which was that it could not be activated. To assay this, we used our Cyclin B1–Cdk1 FRET sensor (Gavet and Pines, 2010a,b).

We measured a sharp increase in FRET signal before NEBD and a subsequent decrease in mitosis in Cyclin B1 ^+/+^ and B1 ^+/−^ embryos injected with Cyclin B1–CaaX (Fig. 7 H). We then assayed whether injecting Cyclin B1–CaaX into Cyclin B1^−/−^ embryos led to any increase in FRET signal and found that there was no detectable increase above background fluctuation (Fig. 7 I). To test whether membrane-tethered Cyclin B1–Cdk1 could be activated if we removed the inhibitory phosphorylation on Cdk1, we inhibited Wee1 by adding MK1775. As expected, adding MK1775 to Cyclin B1^+/+^ and B1^+/−^ embryos caused an almost immediate increase in FRET signal, which subsequently increased at a slower rate than in nontreated cells entering mitosis (∼20% decrease compared with controls, calculated from four independent experiments). This was followed by a noticeably slower decrease after NEBD (Fig. 7 J), which we have also observed in human cells (unpublished data). Adding MK1775 to Cyclin B1^−/−^ embryos expressing membrane-tethered Cyclin B1–RFP–Caax, however, had no effect on the FRET signal (Fig. 7 K). We conclude that tethering Cyclin B1 to the plasma membrane allows it to bind to Cdk1 but that the complex is not detectably activated.

In summary, our results show that Cyclin B1 is essential for mitosis in mammalian cells and that its spatial localization has a crucial role in triggering mitosis at the correct time, even when Cdk1 can be regulated by the antagonistic Wee1–Myt1–Cdc25 pathways.

## Discussion

In this study, we have combined genetic knockouts with live-cell imaging of early mouse embryos to make definitive conclusions concerning the role of Cyclin B1 in the control of cell division in mammalian cells. We show that, unlike other cyclins, Cyclin B1 is essential for division right from the start of development, because embryos lacking Cyclin B1 arrest at the four-cell stage, once they have run out of maternal Cyclin B1. This makes Cyclin B1 unique among the cell cycle cyclins (A1, A2, D1, D2, D3 E1, and E2), all of which are redundant to some degree (Murphy et al., 1997; Liu et al., 1998; Ciemerych et al., 2002; Geng et al., 2003; Kozar et al., 2004; Malumbres et al., 2004; Kalaszczynska et al., 2009). It places Cyclin B1 with Cdk1 as the key essential cell cycle regulators in mammals (Santamaría et al., 2007), at least for embryonic development. There could be a different requirement in somatic cells, as shown for Cyclin A2, which is essential in hematopoietic stem cells, but not in fibroblasts (Kalaszczynska et al., 2009).

The clearly defined arrest at the four-cell stage and our measurement of Cyclin B1 mRNA levels was consistent with the notion that deletion of any essential cell cycle gene should manifest itself soon after the two-cell stage in the mouse, when zygotic genome activation promotes the degradation of maternal mRNA transcripts (Clegg and Pikó, 1983; Bolton et al., 1984; Hamatani et al., 2004; Wang et al., 2004; Lykke-Andersen et al., 2008).

The observation that Cyclin B1–null embryos underwent a second division, even with declining pools of maternal RNA, was consistent with our results obtained in cultured cells that very low amounts of Cyclin B1 were sufficient to trigger entry into mitosis (unpublished data). Several recent siRNA studies in mammalian tissue culture cells have questioned whether Cyclin B1 is required for mitosis, and one suggestion is that Cyclin A2 and Cyclin B2 can compensate for the absence of Cyclin B1 (Bellanger et al., 2007; Soni et al., 2008). This conclusion was somewhat surprising to us given our previous studies in human cells, where we found that Cyclin B2 mainly functions in the disassembly and reassembly of the Golgi apparatus (Draviam et al., 2001). This could reflect a difference in the requirement for Cyclin B1 between embryonic and somatic cells; another possibility is that only small amounts of Cyclin B1 can drive cells into mitosis, and siRNA depletion cannot reduce Cyclin B1 levels below this amount. We show here that only small amounts of Cyclin B1 are indeed able to drive cells into mitosis. We estimate that this threshold is less than 10% of the normal amount of Cyclin B1 in a human epithelial cell and that this is also sufficient to drive human cells into mitosis (unpublished data). This demonstrates the importance of a true genetic null in analyzing the requirement for an enzyme.

Two other strands of evidence also argue against the possibility that Cyclin A2 and Cyclin B2 can compensate for the absence of Cyclin B1. First, in experiments where we treated cells with the Wee1 inhibitor, the activity of the Cyclin A–Cdk and Cyclin B2–Cdk1 complexes should have also increased, but treatment was clearly insufficient to drive entry to mitosis. Second, we found that in the absence of Cyclin B1, cells could not enter mitosis even when the antagonistic phosphatases were inhibited with OA, which, according to current models for the controls on mitotic entry, should have greatly reduced the threshold for kinase activity to drive mitosis (Lindqvist et al., 2009).

In our experiments, we have been able to exploit the power of a genetic null background in embryos by developing a chip for imaging and subsequent genotyping analysis. Thus, we could analyze large numbers of embryos that could subsequently be reliably genotyped and thereby identify statistically significant changes in the timing of cell cycle phases, as well as to characterize in detail cell cycle arrest phenotypes and carry out structure–function analyses. Given the large number of cell cycle regulator mutants that have been generated in the mouse, our system opens up the possibility to combine the power of mouse genetics with time-lapse imaging to determine how the cell cycle is regulated in its natural context.

We have exploited the genetic null background and our imaging chip to clarify the importance of the spatial regulation of Cyclin B1. Cyclin B1 normally shuttles between the nucleus and the cytoplasm such that the great majority of Cyclin B1 is cytoplasmic until it is activated, but the requirement for this nuclear export has been unclear. We find that keeping Cyclin B1 in the cytoplasm by tethering it to the plasma membrane allows it to bind Cdk1 but prevents the complex from being detectably activated, even when there is no Wee1/Myt1 activity. Thus it is likely that Cyclin B1–Cdk1must be able to diffuse in the cytoplasm, or between the cytoplasm and the nucleus, to trigger mitosis in the mouse embryo.

We were surprised to find that a constitutively nuclear Cyclin B1 triggers mitosis too early, even though its partner, Cdk, should still be regulated by the Wee1/Myt1 kinases. Previous studies expressing nuclear Cyclin B1 in the background of endogenous Cyclin B1 in human somatic cells found that nuclear Cyclin B1 only caused premature mitosis when coexpressed with Wee1/Myt1-insensitive Cdk1AF (Jin et al., 1998). Furthermore, our experiments with the MK1775 inhibitor showed that the Wee1/Myt1 kinases are also important in mouse embryos to prevent S phase and G2 phase cells from triggering mitosis. Why cells with nuclear Cyclin B1 cannot prevent premature mitosis is unclear at this point, in part because the nature of the “trigger” to activate Cyclin B1–Cdk1 is undefined. One explanation could be that nuclear Cyclin B1 escapes from a negative regulator, such as Wee1 that is exported to the cytoplasm (Li et al., 2010); alternatively, it could meet an activator, such as Cdc25. It was notable that nuclear Cyclin B1 was only able to shorten G2 phase, which may indicate that S phase and early G2 phase cells still regulate nuclear Cyclin B1–Cdk1. Related to this, we note that in our experiments, constitutively nuclear Cyclin B1 does not interfere with DNA replication. This differs from the results of experiments in *Xenopus* embryos where nuclear B1 can mediate DNA replication. This could reflect a difference between systems or in the properties of the Cyclin B1 used in the earlier study, because it was fused to the N terminus of the S phase–promoting Cyclin E (Moore et al., 2003).

Our results emphasize the importance of spatial control in triggering entry to mitosis, and it will be important to determine the dynamics of the subcellular localization of the essential components of the cell cycle machinery in G2 phase to determine exactly how the timing of mitotic entry is controlled.

## Materials and methods

### Embryo collection and culture

To obtain +/+, +/−, and −/− Cyclin B1 embryos, Cyclin B1 heterozygote females were superovulated with 10 IU of pregnant mare’s serum gonadotropin (Intervet) and 48 h later with 10 IU human chorionic gonadotropin (Intervet) and then mated with Cyclin B1^+/−^ males. The same superovulation protocol was used for the analysis of Cyclin B2. Cyclin B2^−/−^ mice were crossed with Cyclin B1^+/−^, and the resulting B1^+/−^B2^+/−^ mice were crossed to obtain Cyclin B1/B2 double-null embryos. Cyclin B1– and B2–knockout mice (Brandeis et al., 1998) were a gift of T. Hunt. Zygotes were collected into M2 medium and processed as described previously (Jedrusik et al., 2010). Embryos were cultured in KSOM medium (Millipore) under paraffin oil in 5% CO_2_ at 37.5°C. The same culture conditions were used during live imaging. This research has been regulated under the Animals (Scientific Procedures) Act 1986 Amendment Regulations 2012 following ethical review by the University of Cambridge Animal Welfare and Ethical Review Body.

### Genotyping of embryos

Before the diagnostic PCR, a whole-genome amplification step was performed using the illustra GenomiPhi V2 DNA amplification kit (GE Healthcare). Embryos were collected into 5 µl GenomiPhi sample buffer and stored at −20 °C. Before amplification, samples were heated to 95 °C for 5 min and then digested with 5 mg/ml Pronase (Roche) at 55°C for 12 min. The amplification step was performed according the manufacturer’s instructions. All steps before the final PCR were performed in a PCR machine. After amplification samples were purified using the QIAquick PCR purification kit (Qiagen).

For diagnostic PCR, the following 10× buffer stock was used: ammonium sulfate (NH_4_)_2_SO_4_ (166 mM final), Tris, pH 8.8 (670 mM final), MgCl_2_ (67 mM final), and 100 mM β-mercaptoethanol. Samples were run in 50 µl reactions and 3.75 µl DMSO, 0.5 µl Taq Extender Additive (Stratagene), and 0.5 µl Platinum Taq polymerase (Invitrogen) were added per reaction.

The following primer pairs used for genotyping: Cyclin B1: forward, 5′-ATACCTAGCCTGAAACTGAATCCTT-3′; reverse, 5′-GTCCCCAGAAGGTCTTAAAAGTAAC-3′; LacZ: forward, 5′-GCATCGAGCTGGGTAATAAGCGTTGGCAA-3′; reverse, 5′-GACACCAGACCAACTGGTAATGGTAGCGAC -3′. Primer pairs used for genotyping of Cyclin B2: wild-type allele: forward, 5′-CCTTCTCTTAGAATGAGCTGAGTGA-3′; reverse, 5′-ACTAGCCGGGAAGTAAAAGACTG-3′; mutated allele: forward, 5′-AATTCGCCAATGACAAGACG-3′; reverse, 5′-ACTAGCCGGGAAGTAAAAGACTG-3′.

### Plasmids and mRNA microinjection

The coding sequences of the following constructs were cloned into pBluescript RN3P (Zernicka-Goetz et al., 1997) and verified by DNA sequencing: human H2B-RFP, human mRuby-NLS-PCNA, wild-type mouse Cyclin B1–Venus, and human export deficient Cyclin B1^F146A^ with additional SV40 NLS, wild-type mouse Cyclin B1–RFP–Caax (using the following Caax sequence obtained from Clontech: 5′-AAGATGAGCAAAGATGGTAAAAAGAAGAAAAAGAAGTCAAAGACAAAGTGTGTAATTATGTAA-3′), Cdk1 FRET sensor as described previously (Gavet and Pines, 2010a). The sequences of these plasmids are available upon request. mRNAs were transcribed in vitro using the mMessage mMachine T3 kit (Ambion). mRNA working concentrations were 0.3 µg/µl EB3-GFP, 0.01 µg/µl H2B-RFP, 0.1 µg/µl mRuby-PCNA, 0.2 µg/µl Cyclin B1–Venus, and 0.2 µg/µl nuclear Cyclin B1–Venus. Microinjections were performed on an inverted microscope (DM IRB; Leica) using a micromanipulation system (Leica). Embryo holding pipettes were pulled and polished over a Touch-O-Matic Bunsen burner flame from glass capillaries, (GC100-15; Harvard Apparatus) and connected to a hand air pump tube for suction control of embryo holding, using a CellTram pump (Eppendorf). Injection needles were pulled from glass capillaries (GC100TF-10; Harvard Apparatus) on a Flaming Brown Micropipette Puller P-97 (Sutter Instrument). Injection needles were connected to a FemtoJet compressed air injection system (Eppendorf). The needle holder was connected to an Electro 705 electrometer (World Precision Instruments) probe and the embryo holder to an earth wire to create negative capacitance on the embryonic cell membrane to facilitate needle penetration. During injections embryos were held in a drop of M2 medium under mineral oil on a depression glass slide.

### Imaging device

To facilitate embryo handling during high-resolution imaging and recovery after imaging, we developed a multiwell glass chip that contains an array of 252-well chambers open to the medium. This device was developed in collaboration with Dolomite Microfluidics, a microfluidics device manufacturer (part 3200208; Dolomite Microfluidics). Well dimensions were adjusted for standard optical coverslip thickness and embryo stages up to expanded blastocyst. The chip is bonded to a glass reservoir to hold medium. Embryos were loaded into and retrieved from individual chambers with a fine glass pipette. To mount the device onto the microscope, it was inserted into a metal interface (part 3200209; Dolomite Microfluidics) compatible with standard 35-mm Petri-dish microscope stage inserts.

### Imaging

Time-lapse imaging was performed on a confocal spinning-disk microscope system (Intelligent Imaging Innovations, Inc. 3i) comprising an Observer Z1 inverted microscope (Zeiss), a CSU X1 spinning disk head (Yokogawa), and a QuantEM 512SC camera (Photometrics). All live-imaging data shown were collected using a 63× NA 1.2 w.corr. objective (Zeiss), and for most time-lapse acquisitions, time intervals of 8 or 10 min were used. Image acquisition and processing was performed in Slidebook 5 and 6 software. ImageJ was used for additional image processing. Confocal imaging of antibody stained samples was performed on an SP8 confocal microscope system (Leica).

### FRET data analysis

Raw FRET fluorescence intensity data obtained from cytoplasmic and membrane regions was analyzed by loading the transmission and donor channel data (*T(t)* and *D(t)*, respectively) and their corresponding background levels (*δT(t)* and *δD(t)*) into commercially available data analysis software (ORIGIN 9.1; OriginLab). The background levels were subtracted from each channel and the resulting signal normalized to have a maximum and minimum corresponding to 1 and 0, respectively. The background subtracted transmission signal was divided by the background subtracted donor signal to give the FRET signal *f(t)*,

$$f\left(t\right)={\left[\frac{T\left(t\right)-\delta T\left(t\right)}{D\left(t\right)-\delta D\left(t\right)}\right]}_{0}^{1},$$

where ${\left[\ldots \right]}_{0}^{1}$ is the contents normalized with a maximum value of 1 and a minimum of 0. A Fourier filter was applied to *f(t)* to remove known system noise and fluctuations from the final signal.

### Quantitative RT-PCR

Total RNA was extracted from embryos using the Single Cells-to-C_T_ qRT-PCR kit (4458236; ThermoFisher). Reverse transcription was performed after DNase I treatment in the presence of TaqMan oligonucleotides, and subsequent quantitative PCR was performed according to manufacturer’s instructions on a StepOnePlus system (Applied Biosystems). All individual quantitative PCR assays were performed in triplicate. Primer used were mouse ccnb1 (Mm 00838401-g1; ThermoFisher), LacZ (ARXGPZJ; ThermoFisher), and b-Actin (Mm02619580-g1; ThemoFisher). Wild-type F1 control samples were used to normalize Cyclin B1 mRNA and to determine the background levels for LacZ expression. Cyclin B1 and LacZ Ct levels were normalized to the level of mouse b-Actin Ct. Relative RNA levels were calculated using the formula: ΔΔC_T_ = (MeanC_T_, Target − MeanC_T_, Actin)control − (MeanC_T_, Target − MeanC_T_, Actin)Sample. Differences between samples and controls were compared according the 2^(−ΔΔCt) method. The mean and standard error (ΔC_t_ SE) of the mean were determined from three repeats for each sample.

### Western blot analysis

Recombinant mouse His-GST CDK1 and recombinant mouse 10xHis CCNB1Myc (Creative BioMart) were used as protein standards and loaded at 1, 5, 10 ng per lane.

Wild-type mouse embryo samples containing 130 uninjected embryos and 130 embryos injected with CyclinB1–Venus mRNA at the zygote stage were harvested at the two-cell stage, and embryo extracts were prepared using lysis buffer (150 mM KCl, 20 mM Hepes, pH 7.6, 2 mM EGTA, 1.5 mM MgCl_2_, 50 mM NaF, 0.1% NP-40, 10% glycerol, 1 mM Na3VO4, 20 mM β-glycerophosphate, 1 mM dithiothreitol, 10 mM benzamidine HCl, and 25 U/ml Benzonase nuclease [Merck]) supplemented with Protease inhibitor cocktail (P2714; Sigma-Aldrich). Protein samples were separated by SDS-PAGE and analyzed by Western blotting, following standard protocols. Transferred nitrocellulose membranes (Immobilon-P Membrane, IPVH00010; Merck) were blotted with anti-CDK1 antibody (YE324, ab32094; Abcam), anti-Cyclin B1 antibody (EPR17060; ab181593; Abcam) and subsequently with protein A/G-HRP (recombinant protein A/G, peroxidase conjugated; 32490; Pierce). Scanned images were imported into Adobe Photoshop CS6 and the bands were quantified using ImageJ 1.49d (https://imagej.nih.gov/ij/).

### Cdk1 whole-mount antibody staining

Embryos were fixed in 4% PFA in PBS, pH 7.5, for 20 min at RT, washed three times for 10 min in PBS, permeabilized for 20 min in either 0.5% Triton or 0.1% Saponin in PBS, and then washed three times 10 min in either 0.1% Tween 20 or 0.1% Saponin in PBS. Blocking and primary and secondary antibody staining were performed either in 0.1% Tween 20 or 0.1% Saponin with 2% BSA in PBS at 4 °C overnight. After staining, samples were washed three times in PBS and mounted in PBS in a glass-bottom dish for imaging. The primary antibody was mouse monoclonal anti-Cdk1 (1:200, POH-1; ab8040; Abcam), and the secondary antibody was donkey anti–mouse Alexa 488 (1:500; a21202; Invitrogen).

### Inhibitors

OA powder (Calbiochem) was dissolved in water to a stock concentration of 125 µM and diluted in equilibrated KSOM to a final concentration of 250 nM in the imaging device. Wee1 kinase inhibitor (MK1775; Axonmedchem) was diluted in DMSO to a stock concentration of 1 mM and used in equilibrated KSOM at 2 mM and at 20 mM in the FRET experiments in Fig. 7.

### Online supplemental material

Fig. S1 shows the experimental workflow for the experiments and details of the imaging device. Fig. S2 shows representative frames of Videos 3–8 showing the effect of OA and MK1775 treatment on chromosome and microtubule behavior. Fig. S3 compares the time point of arrest of Cyclin B1^−/−^ and B1^−/−^/B2^−/−^ embryos. Fig. S4 quantifies the amount of Cyclin B1 and CDK1 in embryos. Table S1 quantifies the amount of Cyclin B1 mRNA in wild-type and Cyclin B1^−/−^ embryos at the four-cell stage. Videos show differential interference contrast imaging (Video 1) and H2B-RFP imaging (Video 2) of wild-type and Cyclin B1^−/−^ embryos from the two-cell stage up to the blastocyst stage; H2B-RFP (Videos 3 and 5) and EB3-GFP (Videos 4 and 5) imaging of OA-treated embryos; PCNA-RFP imaging Video 6) of wild-type and Cyclin B1^−/−^ embryos; H2B-RFP imaging of MK1775-treated embryos (Video 7); Cyclin B1–Venus imaging (Videos 8–10) and PCNA-RFP (Video 9) or H2B-RFP imaging (Video 10) in Cyclin B1–Venus (Videos 8 and 9), or NLS–Cyclin B1–Venus (Video 10) mRNA-injected blastomeres.

## Supplementary Material

Supplemental Materials (PDF)

Video 1

Video 2

Video 3

Video 4

Video 5

Video 6

Video 7

Video 8

Video 9

Video 10
