# Supplementary material for: Cyclin B1 is essential for mitosis in mouse embryos, and its nuclear export sets the time for mitosis
Source: J Cell Biol. 2018 Jan 2;217(1):179–93. doi: 10.1083/jcb.201612147 (PMC5748970; doi:10.1083/jcb.201612147)
Supplement: Supplemental Materials (PDF) [file JCB_201612147_sm.pdf]

Strauss et al., https://doi.org/10.1083/jcb.201612147

THE JOURNAL OF CELL BIOLOGY

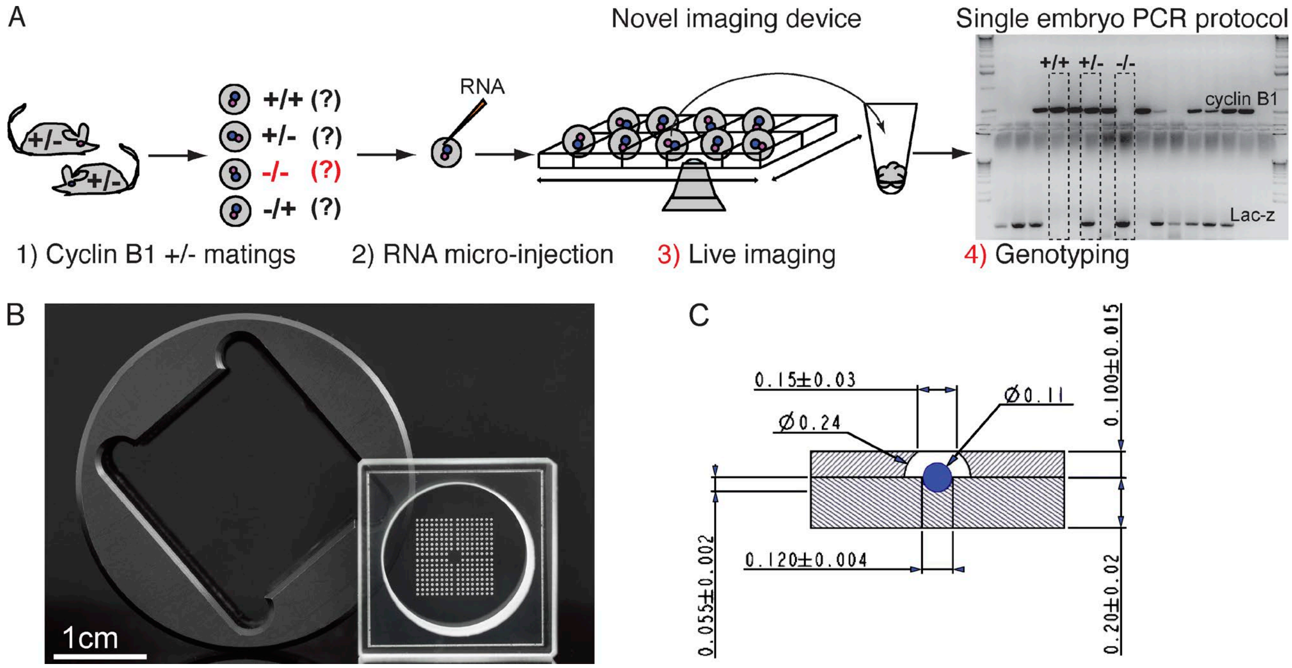

Figure S1. **Experimental setup.** (A) As genotyping could only be performed after time-lapse imaging had been completed, and only 25% of embryos were expected to be Cyclin B1$^{-/-}$, it was important to reduce the loss of embryos during experiments. Therefore, we improved steps 3 (live imaging) and 4 (single-embryo PCR) in our experimental protocol. To make single-embryo genotyping more reliable, we added a whole-genome DNA amplification step before the diagnostic PCR using a Φ polymerase (Materials and methods), because the PCR protocol should give robust results irrespective of the starting amount of DNA (ranging from zygote to 32-cell embryos). To increase the numbers of embryos that can be imaged at high resolution (63×), we developed a multiwell imaging device made of glass in collaboration with a microfluidics company (Dolomite Microfluidics) that allows imaging and subsequent individual recovery of large numbers of embryos. (B) The MI^2 glass imaging chip with 352 holding wells shown in the front, the custom-designed 35-mm microscope adapter in the background. (C) Cross section through the chip showing well geometry and dimensions in centimeters.

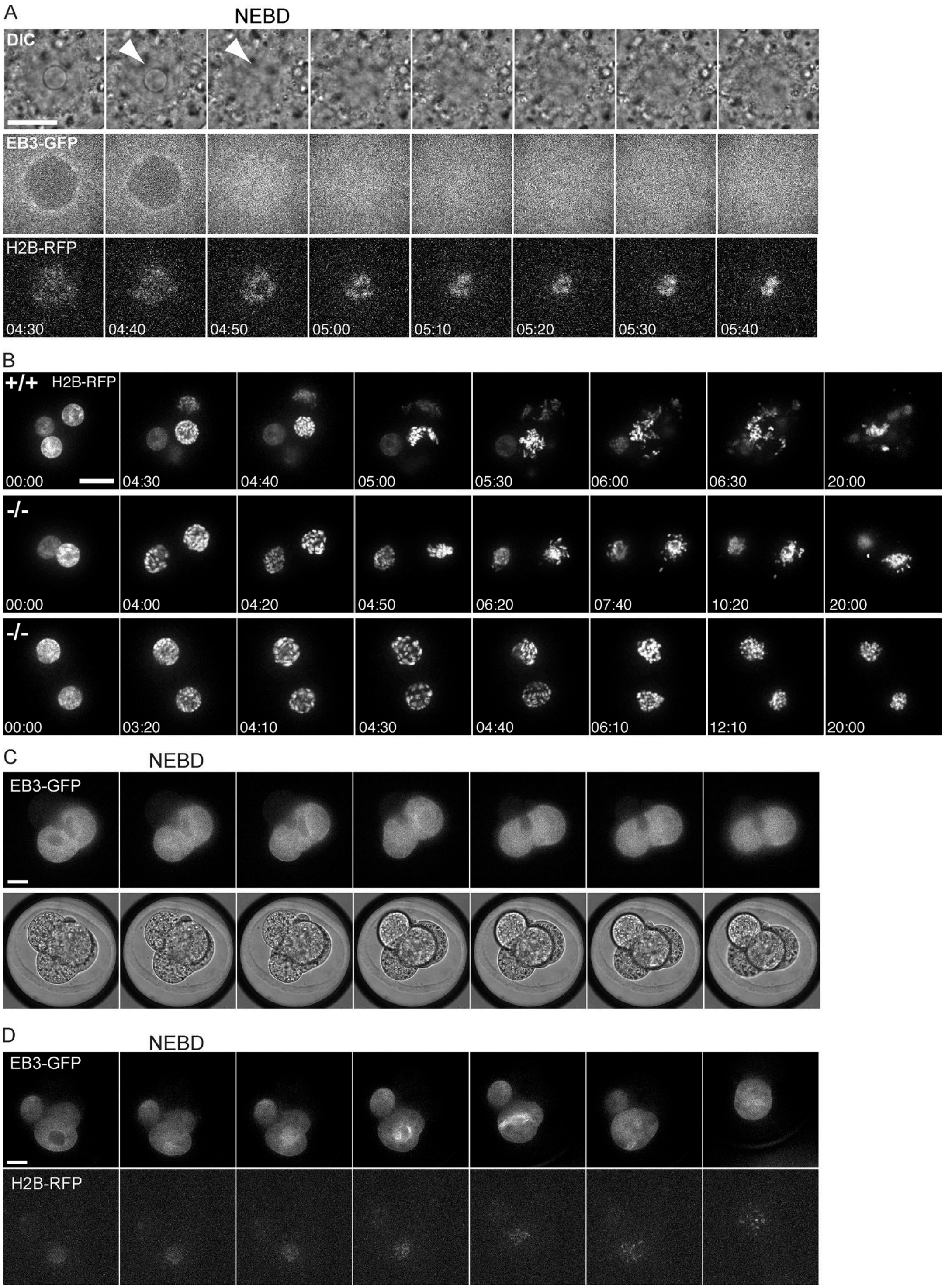

Figure S2. **Cyclin B1–null embryos cannot complete mitotic entry after inhibition of phosphatases.** Mouse oocytes were injected with EB3-GFP and H2B-RFP to visualize tubulin distribution and DNA, respectively. After addition of 250 nM OA, 15 out of 15 oocytes underwent germinal vesicle breakdown. (A) Frames from a representative movie. Although tubulin could clearly enter the nuclear region and DNA condensation was initiated, microtubule bundles or spindles did not form, and DNA did not resolve into chromosomes (bar, 10 µm; time in hours:minutes). Arrowheads indicate disappearance of the nucleolus at NEBD. (B) Representative frames from Video 3. One blastomere of Cyclin B1–null embryos was injected with H2B-RFP mRNA at the two-cell stage and embryos were treated with 250 nM OA at the time when wild-type embryos had started the 8- to 16-cell division. OA was added during the first time point. Top panel shows a wild-type embryo. The middle panel shows a Cyclin B1$^{-/-}$ embryo where individual chromosomes appear to condense and disperse outside the nuclear region (20% of embryos). The bottom panel shows a Cyclin B1$^{-/-}$ embryo where condensation of DNA remains incomplete and confined to a central area (80% of embryos). Bar, 20 µm. (C) Still frames from Video 4, a Cyclin B1$^{-/-}$ embryo injected with EB3-GFP to visualize tubulin distribution shows NEBD after OA addition but no formation of microtubule bundles, corresponding to the nondispersed chromatin phenotype seen with H2B-RFP. Bar, 20 µm. (D) Frames from Video 5 showing a Cyclin B1$^{-/-}$ embryo injected with EB3-GFP and H2B-RFP. NEBD is clearly visible after OA addition, and some larger microtubule bundles form transiently that disperse chromatin, but a normal spindle cannot form. This corresponds to the more dispersed chromatin phenotype seen in the H2B-RFP movies. Bar, 20 µm.

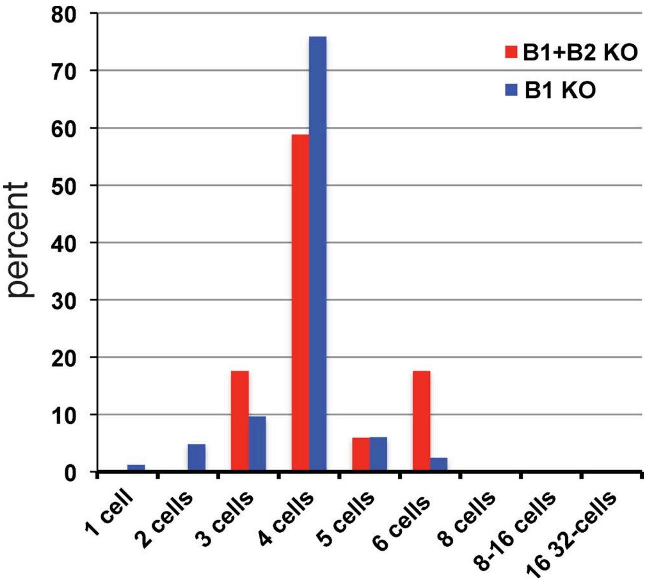

Figure S3. **Cyclin B2 does not appear to contribute to the first divisions.** Comparison of the arrest stages of Cyclin B1/B2–double-knockout embryos (red, *n* = 15, six independent experiments) with Cyclin B1$^{-/-}$ embryos (blue, *n* = 83, 21 independent experiments).

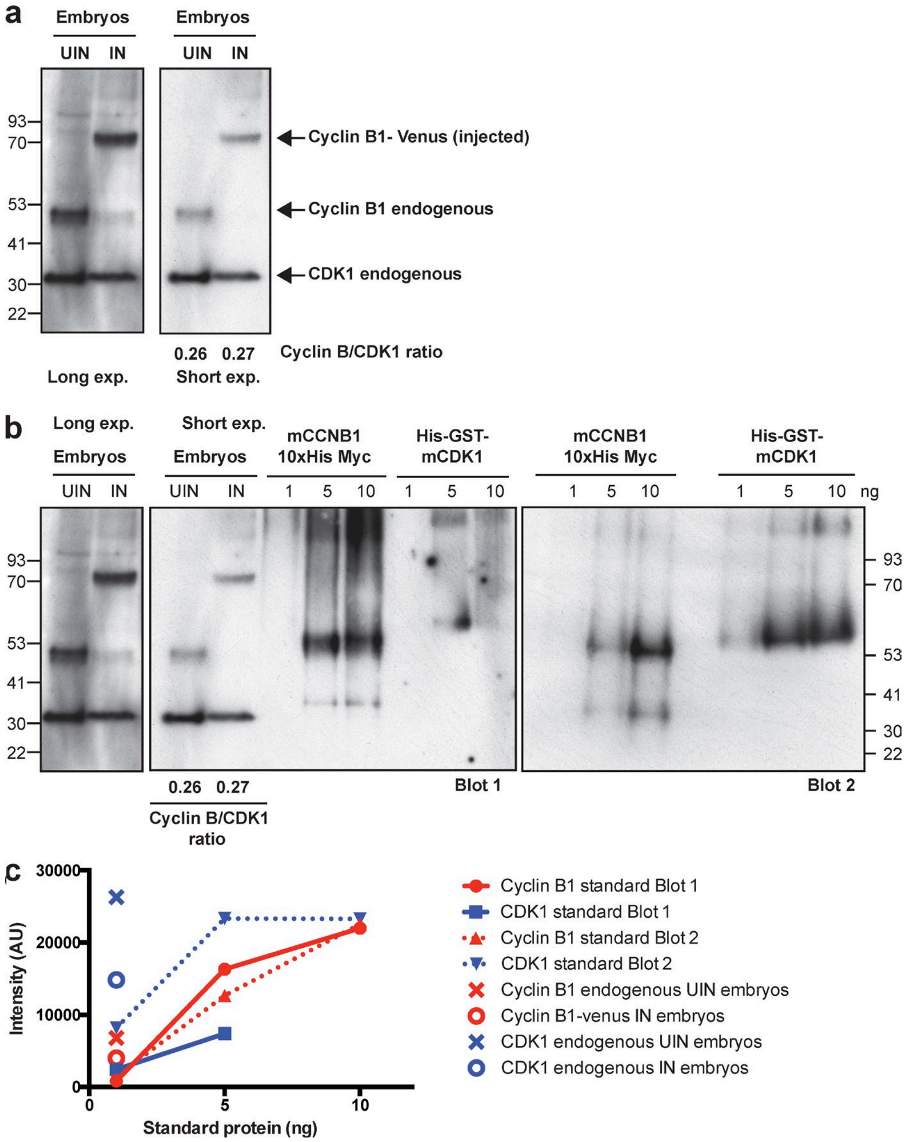

Figure S4. **Injected Cyclin B1 mRNA generates endogenous levels of Cyclin B1 protein.** (A) Western blot showing the levels of endogenous CDK1, endogenous Cyclin B1, and injected Cyclin B1–Venus. Embryo extracts from 130 uninjected (UIN) and 130 Cyclin B1–Venus–injected (IN) wild-type embryos were loaded. The ratios of the band intensities of Cyclin B1/CDK1 were 0.26 and 0.27 for endogenous and injected Cyclin B1, respectively. Longer exposure (left) shows a markedly reduced level of endogenous Cyclin B1 in Cyclin B1–Venus–injected embryos compared with Cyclin B1 levels in uninjected embryos. (B) Western blot shown in A with protein standards of recombinant mouse His-GST CDK1 and recombinant mouse 10xHis Cyclin B1–Myc were loaded at 1, 5, and 10 ng per lane. Because of the quality of the proteins the standards had to be repeated on a separate Western blot. Therefore, the band intensities measured on both blot 1 and blot 2 were plotted in C to show the comparable standard protein ranges on both blots. (C) The intensities of endogenous CDK1 (blue cross or a blue hollow circle), endogenous Cyclin B1 (red cross), and injected Cyclin B1–Venus (hollow red circle) are indicated on the plot.

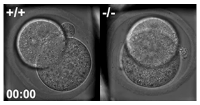

Video 1. **Cyclin B1$^{-/-}$ embryos arrest at the four-cell stage.** Differential interference contrast imaging of wild-type (left) and Cyclin B1$^{-/-}$ (right) embryos shown from the two-cell stage up to the blastocyst stage.

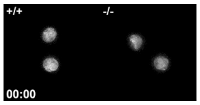

Video 2. **The first two divisions of Cyclin B1$^{-/-}$ embryos are very similar to wild-type divisions.** Wild-type (left) and Cyclin B1$^{-/-}$ (right) embryos injected with H2B-RFP mRNA and shown from the two-cell stage up to the blastocyst stage.

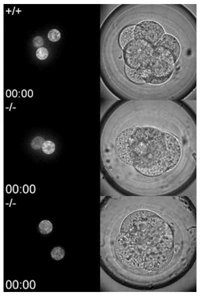

Video 3. **Cyclin B1$^{-/-}$ embryos cannot complete mitotic entry when phosphatases are inhibited with OA.** Wild-type embryo injected with H2B-RFP and treated with OA (top); Cyclin B1–null embryos treated with OA (middle and bottom panels).

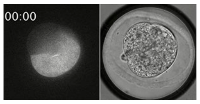

Video 4. **Cyclin B1$^{-/-}$ embryo injected with EB3-GFP showing NEBD after OA treatment.**

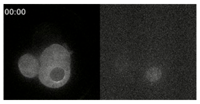

Video 5. **Cyclin B1$^{-/-}$ embryo injected with EB3-GFP and H2B-RFP, showing NEBD and microtubule organization after OA treatment.**

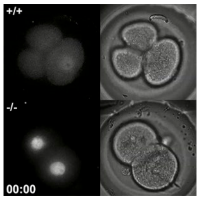

Video 6. **Cyclin B1$^{-/-}$ embryos arrest in G2 after completing S phase.** Wild-type (top) and Cyclin B1$^{-/-}$ (bottom) embryos injected with RFP-PCNA mRNA at the zygote stage. PCNA, left; differential interference contrast, right.

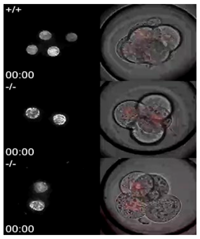

Video 7. **The majority of Cyclin B1$^{-/-}$ embryos cannot complete mitotic entry after Wee1 inhibition.** Wild-type (top) embryo injected with H2B-RFP and treated with Wee1 inhibitor. H2B-RFP, left; transmitted light, right; Cyclin B1$^{-/-}$ embryos, middle, bottom.

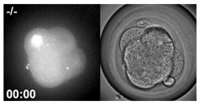

Video 8. **Cyclin B1$^{-/-}$ embryos can be rescued by injecting wild-type cyclin B1 mRNA.** Cyclin B1$^{-/-}$ embryo injected in both blastomeres at the two-cell stage with wild-type Cyclin B1–Venus mRNA, shown from the eight-cell stage. Cyclin B1–Venus, left; transmitted light, right.

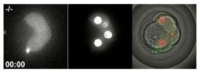

Video 9. **Cyclin B1$^{-/-}$ embryo injected in one blastomere at the two-cell stage with Cyclin B1–Venus and RFP-PCNA mRNA.** Movie shows development of the injected half of the embryo (right) up to the 32-cell stage. Wild-type Cyclin B1–Venus, left; RFP-PCNA, middle; overlay of channels with transmitted light in a central section of the embryo, right.

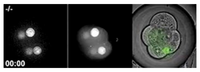

Video 10. **Cyclin B1$^{-/-}$ embryo injected in one blastomere at the two-cell stage with nuclear Cyclin B1–Venus and H2B-RFP mRNA.** Movie shows development of the injected half of the embryo (on the left) up to the 32-cell stage. H2B-RFP, left; nuclear Cyclin B1, middle; overlay of channels with transmitted light in a central section of the embryo, right.

Table S1. **Cyclin B1 mRNA levels in four-cell–arrested embryos and $2^{-\Delta\Delta Ct}$ values for quantitative RT-PCR data**

	Samples	Cyclin B		LacZ	
		$2^{-\Delta\Delta Ct}$	ΔCt SE	$2^{-\Delta\Delta Ct}$	ΔCt SE
Wild type	1	1	0.028619242	0.035605546	0.324969578
	2	1.385005044	0.099038383	0.013003919	0.036394987
	3	0.68458749	0.109598701	0.022691349	0.050573859
	4	1.219938694	0.0823906	0.009454726	0.088479798
	5	2.252591515	0.034353466	0.02394844	0.032934917
	6	1.066659883	0.026175933	0.030077372	0.030197977
	7	1.281661868	0.099038383	0.057488129	0.036394987
	8	1	0.045829181	0.023838291	0.054794099
	9	0.732205072	0.046173081	0.017893691	0.046359091
	10	1	0.038129915	0.01928095	0.046304107
	11	1	0.030451367	0.007225398	0.070394749
8- to 16-cell embryo	12	0.290137648	0.282332291	0.027297571	0.280219194
	13	0.130757612	0.044657926	0.045225952	0.041306844
	14	1.864083015	0.100626876	0.14035853	0.082524005
	15	0.289105858	0.050003501	0.237334463	0.10765287
	16	0.577015845	0.112908808	0.244384727	0.042339172
	17	0.336489792	0.311846363	0.297429549	0.310609568
	18	0.787528007	0.113997549	0.792047107	0.112489616
	19	0.251447659	0.074346706	0.840044153	0.060444232
	20	0.100195113	0.063315489	1	0.051892504
	21	1.066659883	0.11410752	2.05966255	0.128946959
Arrested 4-cell embryo	22	0.000368194	0.066748623	0.160548397	0.015620591
	23	0.000975823	0.058589689	1.618449671	0.035752163
	24	0.001216995	0.071310955	31.73768177	0.019465213
	25	0.001343417	0.057500468	0.408111982	0.058491677
	26	0.001927932	0.044414283	1.117681485	0.050973832
	27	0.002138207	0.032407443	39.23816416	0.023696889
	28	0.003416272	0.045721693	1.151536288	0.047205321
	29	0.003589291	0.063203844	0.504205253	0.033387423
	30	0.004831952	0.066811264	1	0.030419461
	31	0.006796319	0.026789597	0.169861689	0.0339208
	32	0.007537079	0.187563656	4.563509027	0.171027207
	33	0.008000742	0.146884316	5.014358217	0.032961235
	34	0.051724046	0.051082225	157.3769501	0.035925676
	35	1.615160007	0.109598701	7.622214	0.050573859
	36	0.46307767	0.0823906	2.174129118	0.088479798
	37	0.116489171	0.054160479	19.52409486	0.050434981
	38	0.130730416	0.132178762	54.30043195	0.132962783
	39	0.39540622	0.03757972	4.677626509	0.019182496
	40	0.405425789	0.099038383	1.010886613	0.036394987
	41	0.874009752	0.214226871	8.830622749	0.027162391
	42	1.510104472	0.042131393	18.49542964	0.038508227
	43	2.149255049	0.055128818	22.16610699	0.054586427
	44	2.213501752	0.058425288	4.382209795	0.05305053
	45	2.437404533	0.04454514	64.92732473	0.041917946
	46	6.893665643	0.056425179	206.5006398	0.058045545

Quantitative RT-PCR was performed on single embryos to show expression levels of Cyclin B1 or the LacZ-knockout reporter transgene. A control set of wild-type embryos of the B6CBAF1/Crl - F1 strain was collected at the four-cell stage (samples 1–11). Embryos of the Cyclin B1–knockout line used in this study were collected at the time of 8- to 16-cell stage and grouped according to cell numbers: 8–16 cells, samples 12–21; 4–8, arrested cells, samples 22–46. Arrested embryos were pooled from two experiments. As can be seen in column $2^{-\Delta\Delta CT}$ in the arrested group, Cyclin B1 expression is either absent (samples 22–34, three orders of magnitude lower than in wild-type samples 1–11) or corresponds to heterozygous levels, which indicates that some embryos either had residual maternal Cyclin B1 RNA, or were heterozygous arrested due to reasons other than lack of Cyclin B1.
